# Glucose lowering by SGLT2-inhibitor empagliflozin accelerates atherosclerosis regression in hyperglycemic STZ-diabetic mice

**DOI:** 10.1038/s41598-019-54224-9

**Published:** 2019-11-29

**Authors:** Jan Pennig, Philipp Scherrer, Mark Colin Gissler, Nathaly Anto-Michel, Natalie Hoppe, Lisa Füner, Carmen Härdtner, Peter Stachon, Dennis Wolf, Ingo Hilgendorf, Adam Mullick, Christoph Bode, Andreas Zirlik, Ira J. Goldberg, Florian Willecke

**Affiliations:** 1 University Heart Center Freiburg-Bad Krozingen, Cardiology and Angiology I, University of Freiburg, Freiburg, Germany; 2 Ionis Pharmaceuticals, Carlsbad, California USA; 3 Division of Cardiology, Medical University of Graz, Graz, Austria; 4 Department of Medicine, New York University Langone Health, New York, NY USA; 5 Klinik für Allgemeine und Interventionelle Kardiologie/Angiologie, Herz- und Diabeteszentrum Nordrhein-Westfalen, Universitätsklinik der Ruhr-Universität Bochum, Bochum, Germany

**Keywords:** Atherosclerosis, Diabetes complications, Experimental models of disease, Preclinical research, Risk factors

## Abstract

Diabetes worsens atherosclerosis progression and leads to a defect in repair of arteries after cholesterol reduction, a process termed regression. Empagliflozin reduces blood glucose levels via inhibition of the sodium glucose cotransporter 2 (SGLT-2) in the kidney and has been shown to lead to a marked reduction in cardiovascular events in humans. To determine whether glucose lowering by empagliflozin accelerates atherosclerosis regression in a mouse model, male C57BL/6J mice were treated intraperitoneally with LDLR- and SRB1- antisense oligonucleotides and fed a high cholesterol diet for 16 weeks to induce severe hypercholesterolemia and atherosclerosis *progression*. At week 14 all mice were rendered diabetic by streptozotocin (STZ) injections. At week 16 a baseline group was sacrificed and displayed substantial atherosclerosis of the aortic root. In the remaining mice, plasma cholesterol was lowered by switching to chow diet and treatment with LDLR sense oligonucleotides to induce atherosclerosis *regression*. These mice then received either empagliflozin or vehicle for three weeks. Atherosclerotic plaques in the empagliflozin treated mice were significantly smaller, showed decreased lipid and CD68^+^ macrophage content, as well as greater collagen content. Proliferation of plaque resident macrophages and leukocyte adhesion to the vascular wall were significantly decreased in empagliflozin-treated mice. In summary, plasma glucose lowering by empagliflozin improves plaque regression in diabetic mice.

## Introduction

Despite recent advances in the understanding of its pathophysiology and therapy, diabetes continues to be a major risk factor for the development of atherosclerosis and its complications such as coronary heart disease, stroke and peripheral artery disease. In advanced atherosclerotic disease, alleviating the atherosclerotic plaque burden has been achieved by lowering plasma cholesterol levels^1^. Intravascular ultrasound studies show some reduction in plaque volume, albeit a few percent over 1–2 years in non-diabetic patients^2,3^. However, the presence of diabetes partially negates atherosclerosis regression in both humans and mice^4,5^. The reasons for the defective regression in patients with diabetes are unclear and whether this is due to defective insulin signaling, hyperglycemia or aggravated hyperlipidemia is subject of ongoing research. Recently, the glucose-lowering sodium-glucose-cotransporter 2 (SGLT2) inhibitor empagliflozin has shown beneficial actions on all-cause and cardiovascular mortality in diabetic subjects^6^. The SGLT2 is expressed on the epithelial lining of the proximal convoluted tubule in the kidneys. Inhibition of SGLT2 prevents the reuptake of glucose from the glomerular filtrate, lowers the glucose level in the blood and stimulates the excretion of glucose in the urine. While the pharmacology of SGLT2 inhibition is well understood, the mechanisms for its cardiovascular benefits are currently investigated: One proposed mechanism is the beneficial effect of glucose reduction on inflammatory processes in atherosclerosis.

Atherosclerosis regression does not involve the same mechanisms as progression in reverse order. There are distinct cellular and molecular processes that mobilize plaque elements to resolve the plaque. Regressive plaques are characterized by reduced overall macrophage content and increased fibrotic material, less apoptosis and smaller necrotic cores. Overall, the combination of these features is suggestive of plaques of increased stability and less susceptibility to rupture and subsequent thrombosis formation in humans^7^. A complete understanding of these processes requires the development of suitable mouse models and selection of appropriate study protocols^7^. We have recently developed a novel mouse model of atherosclerosis progression and regression using oligonucleotide regulation of the LDL receptor (LDLR)^8^. We now used inhibition of the LDLR and the scavenger receptor B1 (SRB1) to achieve higher plasma cholesterol levels and more advanced atherosclerotic lesions. Hepatic expression of the HDL receptor SRB1 plays an important role in reverse cholesterol transport, the transport via HDL of cholesterol from peripheral tissues (e.g. atheromatous plaques) to the liver for recycling or biliary excretion^9^.

In the current study we now make use of this model to study the effect of SGLT-2 inhibitor-mediated glucose lowering on atherosclerosis regression in a STZ-induced diabetes mouse model.

## Material and Methods

### *In vivo* studies

#### Atherosclerosis regression study

**Figure 1:**
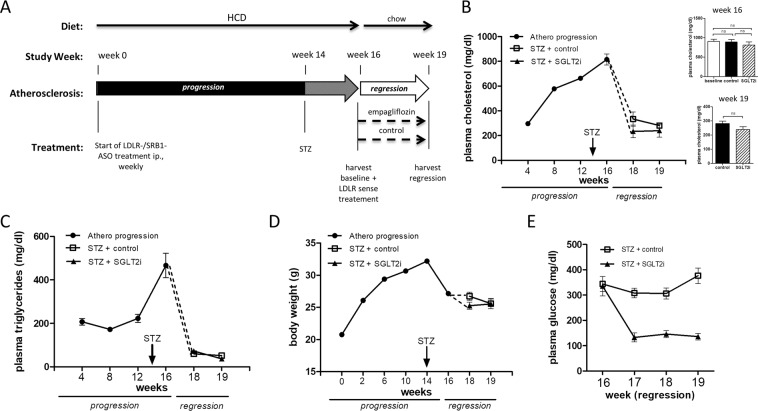
Applying antisense/sense oligonucleotides and the SGLT2 inhibitor empagliflozin to regulate plasma cholesterol and glucose levels. (**A**) Timeline of atherosclerosis regression study. Wildtype mice received weekly ip. injections of LDLR-/SRBI- antisense and HCD during the atherosclerosis *progression* period and were subjected to five consecutive STZ-injections at week 14. An atherosclerosis baseline group was harvested in week 16. Atherosclerosis regression was then initiated by LDLR sense treatment and switching to chow diet. All mice received either the SGLT2 inhibitor empagliflozin or vehicle. **(B)** Total plasma cholesterol during atherosclerosis progression and regression, inlets on the right show plasma levels at 16 weeks and 19 weeks. **(C)** Total plasma triglyceride levels during atherosclerosis progression and regression. **(D)** Body weight and **(E)** 4-hour fasting plasma glucose after STZ-treatment (n = 8–11/group). ns = not significant. Error bars represent SEM.

Male wild type C57BL/6J mice were acquired from Janvier (Janvier Labs, Le Genest-Saint-Isle, France). To induce atherosclerosis progression all mice received weekly intraperitoneal (i.p.) injections of LDLR and SRB1 antisense oligonucleotides (ASO) for 16 weeks (see Fig. 1A for study timeline). In addition all mice received high cholesterol diet (HCD) with 1.25% cholesterol for 16 weeks (ssniff GmbH, Soest, Germany, EF D12108). At study week 14, all mice received i.p. injections of streptozotocin (STZ, 50 mg/kg body weight, on five consecutive days). Only mice with 4-hour fasting glucose levels >250 mg/dl ten days after the final STZ injection were classified as diabetic and were included into the study. After study week 16 a baseline group was harvested to assess baseline progressive atherosclerosis. In order to lower plasma cholesterol in the remaining mice we switched from HCD to chow diet and replaced the i.p. LDLR/SRB1 antisense oligonucleotide injections by LDLR sense oligonucleotides (SO) at regression week one and three. During the entire three week regression period mice received either the SGLT2 inhibitor empagliflozin or normal drinking water. After three weeks all remaining mice were harvested for assessment of atherosclerosis regression. The experimental protocols were approved by the animal ethics committee of the University of Freiburg and the regional board of Freiburg, Germany and were carried out in accordance with institutional guidelines.

#### Intravital microscopy study

To determine how changes in circulating levels of glucose affected adherence of circulating leukocytes to endothelial cells, we performed intravital microscopy of abdominal venules. At age 6 weeks STZ-diabetes was induced. Mice with 4-hour fasting glucose levels >250 mg/dl ten days after the final STZ injection were considered diabetic and were included into the study. After day 10, mice received either the SGLT2 inhibitor empagliflozin (35 mg/kg body weight per day) or normal drinking water for one week. After one week of empagliflozin treatment, intravital microscopy was performed. 4 hours prior to surgery all mice received an intraperitoneal injection of 0.2 µg TNF-α to stimulate leukocytes adhesion to the endothelial lining (Recombinant Mouse TNF-α (aa 80-235) Protein, Cat. 410-MT-010, R&D Systems, Wiesbaden, Germany, diluted in 200 µl PBS). All mice were anesthetized by i.p. injection of ketamine (Inresa, Freiburg, Germany, #07714091) and xylazine hydrochloride (Rompun 2%, Bayer Vital GmbH, Leverkusen, Germany, #1320422). All mice received a retroorbital injection of 60 µl rhodamine (C = 1 mg/ml, diluted in PBS, Rhodamine 6 G, R4127, Sigma-Aldrich Chemie GmbH, Steinheim, Germany). After disinfection of the abdominal area, the peritoneum was opened and the mesenteric vessels were exposed. Intravital microscopy was then implemented by using a fluorescence microscope (Axiotech Vario 100 HD, Carl Zeiss Microscopy GmbH, Göttingen, Germany). For intravital microscopy terminal venules were located and videos with a length of 30 s were taken (10 videos per mouse). An area with a length of 200 µm and a width of 100 µm was marked and rolling and adhering leukocytes were counted. The result was normalized to the leukocyte numbers measured in each animal before surgery. All analysis of adhering and rolling leukocytes were done by blinded investigators with the software Zen 2.3 lite.

#### Antisense-/sense oligonucleotide treatment

LDLR and SRB1 ASO were injected intraperitoneally (10 mg/kg and 30 mg/kg body weight respectively) once a week for 16 weeks. GalNAc-conjugated sense oligonucleotides (SO) designed to bind and inactivate the LDLR ASO were injected intraperitoneally at the beginning of week 1 and week 3 of the regression period (20 mg/kg body weight). All antisense/sense oligonucleotides were diluted in sterile PBS. GalNAc-conjugated Gen 2.5 ASO targeting mouse LDLR and non-GalNAc-conjugated ASO Gen 2.0 targeting SRB1 were developed and provided by Ionis Pharmaceuticals (Carlsbad, CA, USA).

#### STZ injections

For inducing STZ diabetes the low-dose strategy with 5 STZ-injections (50 mg/kg body weight) on 5 consecutive days was used, according to the protocol of the Diabetes Complications Consortium^10^. STZ (STZ ≥ 75% α-anomer basis, ≥98% (HPLC) powder S0130-500mg), sodium citrate dihydrate (W302600-1KG-K) and sodium citrate monohydrate (71497-250G) were all acquired from Sigma-Aldrich Chemie GmbH (Steinheim, Germany).

#### Empagliflozin treatment

Empagliflozin was diluted in the drinking water (0,073 mg/ml) and given at a dose of 35 mg/kg body weight per day; at a similar dose range reported by others^11,12^. Empaglifozin was given in the drinking water to replicate the route of intake in humans and to avoid the stress associated with parenteral administration. For appropriate dosing, daily drinking water consumption of STZ-diabetic mice was measured in a previous experiment. Empagliflozin was a kind gift of Boehringer-Ingelheim (Ingelheim, Germany).

#### Blood collection

Blood was collected after 4 hours of fasting from the retro-orbital plexus of narcotized mice using heparinized micro capillary tubes. Blood was centrifuged at 500 g for 5 minutes for removal of cells. Plasma was then used for lipid measurements and/or frozen at −80 °C.

#### Cholesterol assay

Total plasma cholesterol levels were quantified by photometric assay consisting of a reagent (Cholesterol FS, REF 113009910026) and a standard control (Cholesterol Standard FS, REF 113009910030, both Diagnostic Systems GmbH, Holzheim, Germany), according to the manufacturer’s instructions.

#### Triglyceride assay

Triglyceride plasma levels were analyzed by photometric assay consisting of a reagent (Triglycerides FS, REF 157609910021) and a standard control (Triglycerides Standard FS, REF 157003010030, both Diagnostic Systems GmbH, Holzheim, Germany), according to the manufacturer’s instructions.

#### Blood glucose measurement

Blood was obtained via puncture of the tail vein after 4 hours of fasting. The first blood drop was discarded. Blood glucose levels were measured by a glucometer (Model NC, REF 06870333001) and test strips (Product No.: 6114963, both Roche Diabetes Care Deutschland GmbH, Mannheim, Germany).

#### Leukocyte quantification

Total leukocytes were quantified by forward/sideward scatter in the clinical laboratory of the University Hospital Freiburg.

#### Flow cytometry

Flow cytometry to assess leukocyte subgroups was implemented with 100 µl of whole-blood. First, erythrocytes were lysed by incubating the blood with RBC lysis buffer (Cat.: 420301, BioLegend Biozol, Eching, Germany) for 4 minutes at room temperature (RT). The lysis was stopped by addition of PBS (Cat.No. P04-36500, Pan-Biotech GmbH, Hilden, Germany), a cell pellet was spun down at 350 g for 5 minutes and the supernatant was discarded. The lysis was repeated 2–3 times. Then the pellet was resuspended in 1 ml FACS-buffer (PBS, 0.5% Bovine Serum Albumin (Albumin Fraction V, A1391,0050, PanReac AppliChem, Barcelona, Spain), 1% Fetal Bovine Serum). Extracellular surface proteins were stained by incubating the leukocytes diluted in FACS-buffer with the fluorescing antibodies for 30 min in the dark at 4 °C.

The antibodies that were used are CD45.2-Pacific Blue (REF 48–0454, eBioscience, Affymetrix Inc., San Diego, USA), CD11b-APCCy7 (Cat. 557657, BD Bioscience, San Jose, USA), CD115-PE (REF 12–1152, eBioscience, invitrogen, Thermo Fisher Scientific, Affymetrix Inc., San Diego, USA), Ly6C-PerCP (Cat. 128012, BioLegend, London, GB), CD3e-FITC (REF 11–0031, eBioscience, San Diego, USA), CD3-V500/Amcyan (Cat. 560771 BD Bioscience, San Jose, USA), CD4-FITC (Cat. 100510 BioLegend, London, GB), CD8-PerCP (Cat. 100739 BioLegend, London, GB), CD25-PE (Cat. 12-0251-82, eBioscience, San Diego, USA), Ly6G-APC (Cat. 127614 BioLegend, London, GB), CD19-APCCy7 (Cat. 557655 BD Bioscience, San Jose, USA), CD19-PECy7 (REF 25–0193 eBioscience, invitrogen, Thermo Fisher Scientific, Affymetrix Inc., San Diego, USA) (all anti-mouse).

The intracellular protein FoxP3 was stained with a FoxP3 staining kit (Cat. 00-5523-00, eBioscienceTM Foxp3 / Transcription Factor Staining Buffer Set, Invitrogen, Thermo Fisher Scientific, Life Technologies Corp., Carlsbad, USA) according manufacturer’s instructions The samples were analysed with a fluorescence-activated cell sorter (FACS, BD FACSCanto^TM^ II, BD Biosciences, Heidelberg, Germany). For compensation and analysis the software FlowJo was used.

### Histological and morphometric analysis of aortic roots

#### Harvest of aortic roots

Mice were anesthetized by intraperitoneal injection of xylazine hydrochloride (0.02 mg/g body weight, Rompun 2%, Bayer Vital GmbH, Leverkusen, Germany, #1320422) and ketamine (0.3 mg/g body weight, Inresa, Freiburg, Germany, #07714091). After opening of the abdomen and thorax, the aorta was flushed with PFA4% (Roti®-Histofix 4%, No. P087.3, Carl Roth GmbH & Co.KG Karlsruhe, Germany) through the left ventricle. The aortic root was cut from the heart and stored in PFA4% for 60 min at RT. The aortic root was washed with PBS three times for 10 minutes and then incubated consecutively into sucrose 10%, 20% and 30% over night at 4 °C (Saccharose, CAS 57-50-1, Merck, Darmstadt, Germany). After sucrose incubation the roots were embedded in O.C.T. (Tissue-Tek® O.C.T Compound, Sakura Finetek Europe B.V., Alphen aan den Rijn, Netherlands). To quantify plaque size and plaque composition, aortic roots were sectioned in 6μm sections on a cryostat (CM 1510 S Leica Microsystems Nussloch GmbH, Nussloch, Germany) at −20 °C and stained as previously described^13,14^. Sections of the aortic root underwent analysis for the total wall area (=intima + media), intimal lesion area (intima), and medial area (media) as described previously. For quantification of total plaque size four sections per animal at 0 µm, 32 µm, 72 µm and 112 µm were analyzed. Plaque composition, the percentage of positively stained area for lipids (Oil Red O), macrophages (anti-mouse CD68), and collagen (Picrosirius red) was quantified and calculated by blinded investigators using computer-assisted image analysis software (Image Pro, Media Cybernetics, Rockville, MD, USA). All animal-related procedures were approved by the Animal Care Committee of the University of Freiburg and all mice were housed under specific pathogen-free conditions.

#### Oil Red O (ORO) staining

Staining of lipids was done as previously described by our group^13^: An Oil red O solution (0.5%) was prepared a day before the staining procedure. 2.5 g pure Oil red O (Sigma-Aldrich, St. Luis, MO, USA, #O0625) were dissolved in 500 ml propylene glycol (1,2-Propandiol, Fisher Scientific, Waltham, MA, USA, #S25769) at 95 °C. The suspension was then filtered through 185 mm filter paper (Whatman GmbH, Dassel, Germany, #10314714) and cooled down to RT. Prior to staining, filtration was repeated with a 0.2 μm filter (Nalgene vacuum filtration system, Sigma-Aldrich, St. Luis, MO, USA, #568-0020). Sections of the aortic root and arch were fixed for 10 min in 10% formalin. After rinsing with water slides were dehydrated in 100% propylene glycol. Sections were stained in 0.5% Oil Red O for 25 min at 60 °C. After 10 min rinsing with water cell nuclei were counterstained for 5 sec in 25% hematoxylin (Sigma-Aldrich, St. Luis, MO, USA, #H3136-100G) and dipped in 0.25% ammonium (Ammonia water 0.25%, Electron Microscopy Sciences, Hatfield, PA, USA, #26123-10). All sections were embedded in glycerol gelatin (Sigma-Aldrich, St. Luis, MO, USA, #GG1) and covered with a cover slip.

#### Picrosirius red staining

As previously described^13,15^, for immunohistological staining of collagen a sirius red solution (0,1%)(Polyscience inc., Warrington, PA, USA, #09400) was prepared in saturated aqueous picric acid (Ricca Chemical Company, Arlington, TX, USA, #5860-32) and filtered. After desiccation sections were fixed in 10% buffered formalin for 10 min at RT. After rinsing with tap water, the slides were incubated for 3–4 hours in picrosirius red solution. Slides were rinsed twice for 1 min in 0.01 N HCl and dehydrated in ascending concentrations of Ethanol (70% ethanol for 30–45 sec, 95% ethanol about 5 min, and 100% ethanol about 5 min) and xylenes (5 min).

#### Anti-CD68 staining

Immunohistochemical staining of CD68 was done as previously described^13,15^. Aortic root sections were equilibrated to RT and fixed in acetone for 9 min. To avoid leakage of staining roots were framed with polysiloxane (15% Dimethylpolysiloxane, Sigma-Aldrich, St. Luis, MO, USA, #DMPS-12M), 84% propanol (Isopropyl-alcohol, VWR International GmbH, Darmstadt, Germany, # VW3250-4) 1% H_2_SO_4_ (Fisher Scientific, Schwerte, Germany, # A300-500) at 65 °C. Sections were incubated in 0.3% H_2_O_2_ (EMD Chemicals, Merck KGaA, Darmstadt, Germany, # HX0635-1) for 30 min at RT. After repeated rinsing with PBS for 5 min at RT, sections were incubated with 50 μl 10% rabbit serum (Vector Laboratories, Burlingame, CA, USA, #S-5000) for 30 min, to avoid unspecific binding. According to the manufacturer’s protocol, serum was removed and staining was accomplished with the primary antibody (1:500; anti-CD68 (rat anti-mouse), BioRad, Puchheim #MCA1957GA) for 1 hour at RT. After three times rinsing with PBS for 5 min at RT, sections were incubated with a biotinylated rabbit anti-Rat (1:200, Vector Laboratories, Burlingame, CA, USA, #BA-4001) for 30 min at RT. This step was followed by an additional 3 times rinsing with PBS for 5 min at RT. Sections were subsequently incubated with 50 μl of Elite PK-6100 Vectastain ABC kits (Vector Laboratories, Burlingame, CA, USA, #PK-6100) for 30 min at RT. After washing with PBS for 5 min at RT, staining was developed with ImmPACT AMEC Red Substrate (Vector Laboratories, Burlingame, CA, USA, #SK-4285) for 1 to 3 min. Stained sections were washed for 20 min with rinsing water and cell nuclei were counterstained with hematoxylin.

#### Ki67/CD68/DAPI immunofluorescence stain

After fixation (PFA4% for 10 min) and permeabilization (Triton X-100 0.5% (No: A4975,0100, PanReac AppliChem, Barcelona, Spain) with Tween20 0.25% (No: 142312.1611, PanReac AppliChem, Barcelona, Spain) in PBS for 5 min) slides were incubated in goat serum 10% (Normal Goat Serum Blocking Solution, Cat: S-1000, Vector Laboratories, Peterborough, GB) for 1 hour. For Ki-67 immunofluorescence staining, slides were incubated with anti-Ki67 rabbit primary antibody (ab16667, abcam, Cambridge, GB) over night at 4 °C followed by secondary anti-rabbit goat biotin-conjugated antibody (Cat: BA-1000, Vector Laboratories, Peterborough, GB) for 45 min. Then Fluorescein Avidin DCS (Cat: A-2011, Vector Laboratories, Peterborough, GB) was added. Slides were incubated with rabbit serum 10% (Normal Rabbit Serum Blocking Solution, Cat: S-5000, Vector Laboratories, Peterborough, GB) for 1 hour. For CD68 immunofluorescence staining, slides were incubated with anti-CD68 rat antibody (Rat Anti Mouse CD68, MCA1957GA, BioRad, Puchheim, Germany) for 1 hour as first antibody and afterwards with anti-rat rabbit biotin-conjugated antibody (Cat: BA-4001, Vector Laboratories, Peterborough, GB) for 45 min as the second antibody. Then Texas Red Avidin DCS (Cat: A-2016, Vector Laboratories, Peterborough, GB) was added. Sections were then covered with DAPI mounting medium (Fluoroshield Mounting Medium with DAPI, ab104139, abcam, Cambridge, GB). Pictures were taken with a fluorescence microscope and the ratio of triple positive (Ki67/CD68/DAPI) versus double positive cells (CD68/DAPI) were counted as previously described^16^.

### Statistical analysis

Student’s t-test was used for comparing 2 groups. One-way ANOVA and Newmann-Keuls post hoc test were used for comparing 3 groups. Results with α < 0.05 were considered statistically significant. Statistics and graphs were created with GraphPad Prism. All data are expressed as the mean ± SEM.

## Results

### Application of antisense/sense oligonucleotides and the SGLT2 inhibitor empagliflozin to modulate plasma cholesterol and glucose levels in mice

To induce atherosclerosis, we treated wildtype C57BL/6J mice with weekly injections of ASOs against the *ldlr* and *srb1* mRNA (Fig. 1A). ASO treatment decreased hepatic Ldlr and Srb1 expression by 12- and 10-fold, respectively (Supplementary Fig. 1). We used a combination of two ASOs and a HCD to induce severe hypercholesterolemia (>600 mg/dl), comparable to commonly used knock-out mouse models of atherosclerosis. After additional induction of STZ-diabetes, plasma cholesterol and plasma glucose increased to 862 ± 37 mg/dl and 356 ± 22 mg/dl, respectively at 16 weeks (Fig. 1B). After sacrifice of a baseline (atherosclerosis progression) group, plasma cholesterol was decreased by switching to a chow diet and treatment with a LDLR sense oligonucleotide. Treatment of one group of diabetic mice with the SGLT2 inhibitor empagliflozin normalized plasma glucose levels to 140 ± 14 mg/dl while vehicle treated mice continued to be hyperglycemic (Fig. 1E). Notably, there was no difference in plasma cholesterol in the empagliflozin- compared to the vehicle-treated group, excluding a possible bias on regression by different cholesterol levels (week 19, Fig. 1B, insert). Plasma triglycerides increased significantly after induction of STZ-diabetes and decreased after LDLR sense treatment and diet switch to levels commonly found in wildtype mice (Fig. 1C). Similar to plasma cholesterol, triglycerides were not affected by empagliflozin compared to vehicle. As described previously^17^, induction of STZ-diabetes caused a weight loss in all mice. (Fig. 1D).

### Empagliflozin treatment leads to diminished atherosclerotic plaque size compared to vehicle treated hyperglycemic mice

**Figure 2:**
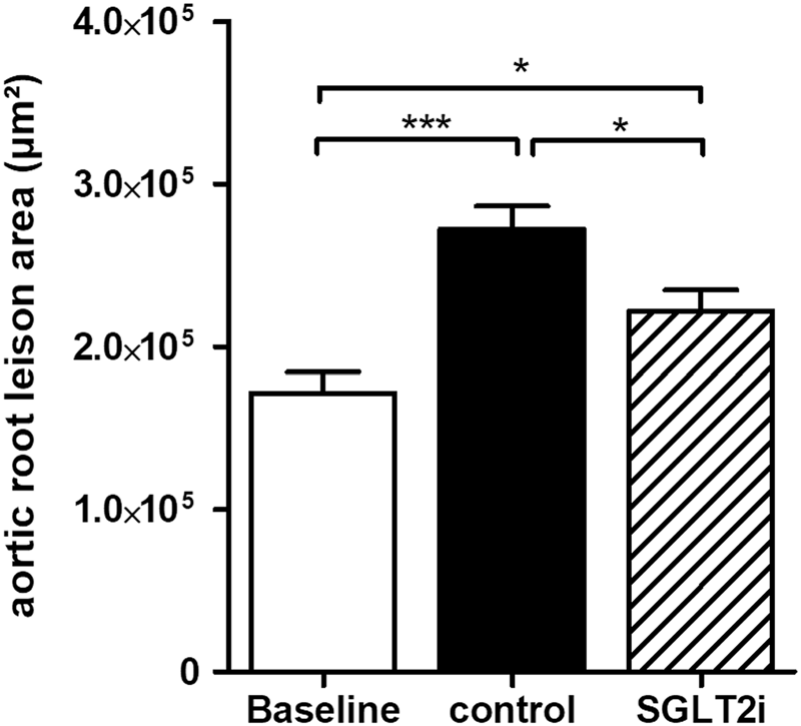
Diminished atherosclerotic plaque size in SGLT2 inhibitor-treated mice. Mean of aortic root lesion area, n = 7–9/group. *p < 0.05, ***p < 0.001. Error bars represent SEM.

Mice were sacrificed either at baseline (16 weeks of atherosclerosis progression) or after additional 3 weeks of cholesterol lowering with or without empagliflozin treatment to assess atherosclerosis progression and regression. After 16 weeks of hypercholesterolemia, mice in the baseline group displayed substantial atherosclerotic plaques in the aortic roots. Plaque size increased further in hyperglycemic mice over the next three weeks (Fig. 2). However, atherosclerotic lesion size was significantly less in SGLT2-inhibitor treated, normoglycemic mice after three weeks—2.7 × 10^5^ ± 1.4 × 10^4^ µm^2^ (control) vs. 2.2 × 10^5^ ± 1.3 × 10^4^ µm^2^ (empagliflozin). Therefore, glucose reduction slowed the rate of progression.

### SGLT2-inhibitor treatment accelerates features of plaque stability during atherosclerosis regression

**Figure 3:**
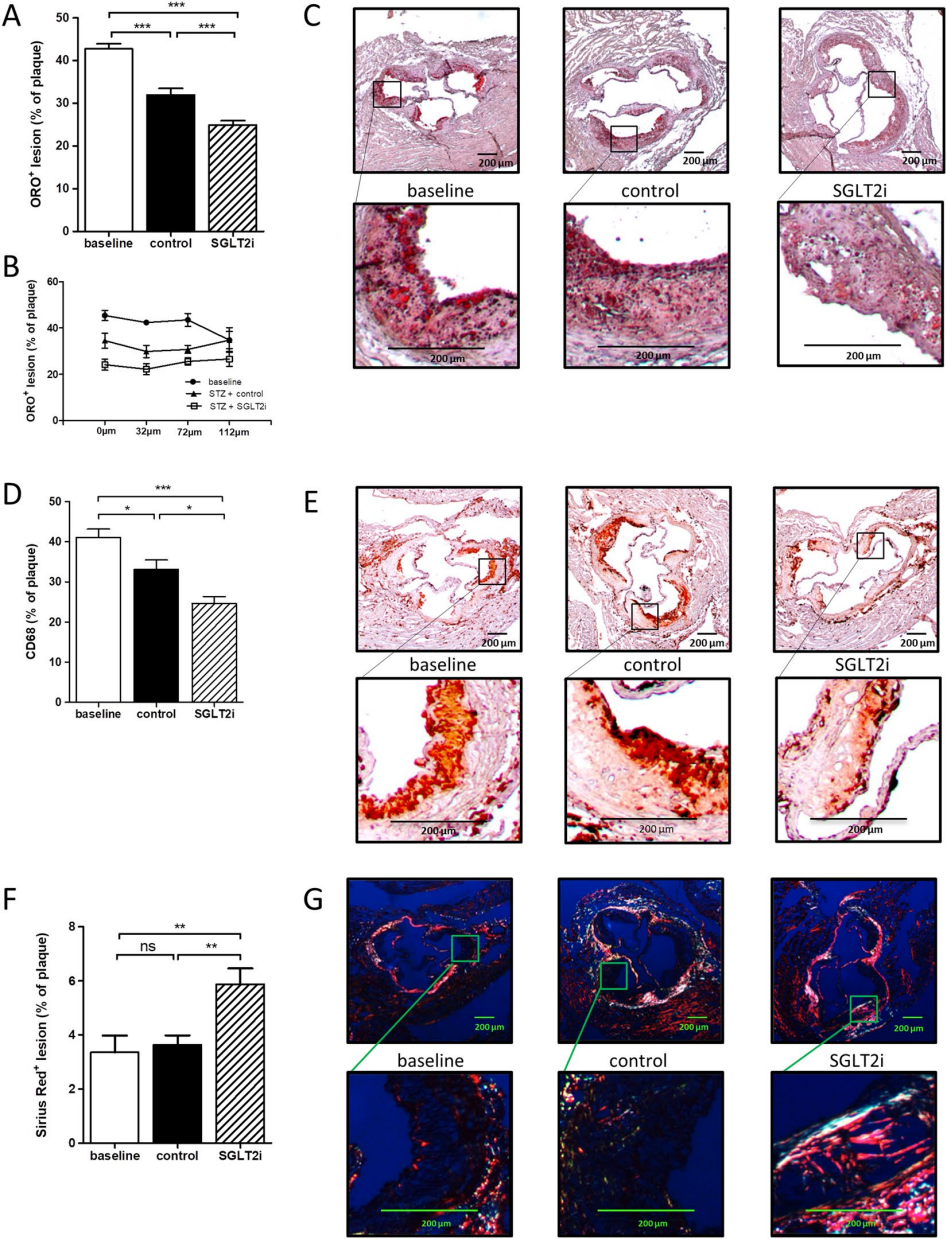
SGLT2-inhibitor treatment accelerates features of plaque stability during atherosclerosis regression. (**A)** Quantification of lipid content of aortic root plaques by ORO-staining, 4 sections/mouse, n = 7–9. **(B)** Lipid content at 0 µm, 32 µm, 72 µm, 112 µm. **(C)** Representative pictures of ORO-stained aortic root sections. **(D)** Quantification of CD68^+^ macrophages in aortic root sections. 1 section/mouse, n = 7–9/group. **(E)** Representative pictures of respective aortic root sections and magnifications, n = 7–9/group. *p < 0.05, ***p < 0.001. Error bars represent SEM. **(F)** Quantification of collagen content of aortic root plaques by Picrosirius-Red staining, 2 sections/mouse, n = 7–9. **(G)** Representative pictures of Picrosirius-Red-stained aortic root sections. **p < 0.01. ns = not significant. Error bars represent SEM.

Regression can lead to decreased lesion size, but more obviously alters cellular plaque composition to a more stable morphology. Both atherosclerosis regression groups showed a significant decrease in lipid deposition and CD68^+^ macrophages compared to the baseline group (Fig. 3A–E). However, both lipid and CD68^+^ macrophage content was further decreased in normoglycemic, empagliflozin-treated mice by ~25% compared to hyperglycemic mice. Concomitantly, content of plaque-stabilizing collagen was increased by ~60% in empagliflozin treated mice (Fig. 3F,G).

### Leukocytes and leukocytes subsets are not affected by empagliflozin during atherosclerosis regression

**Figure 4:**
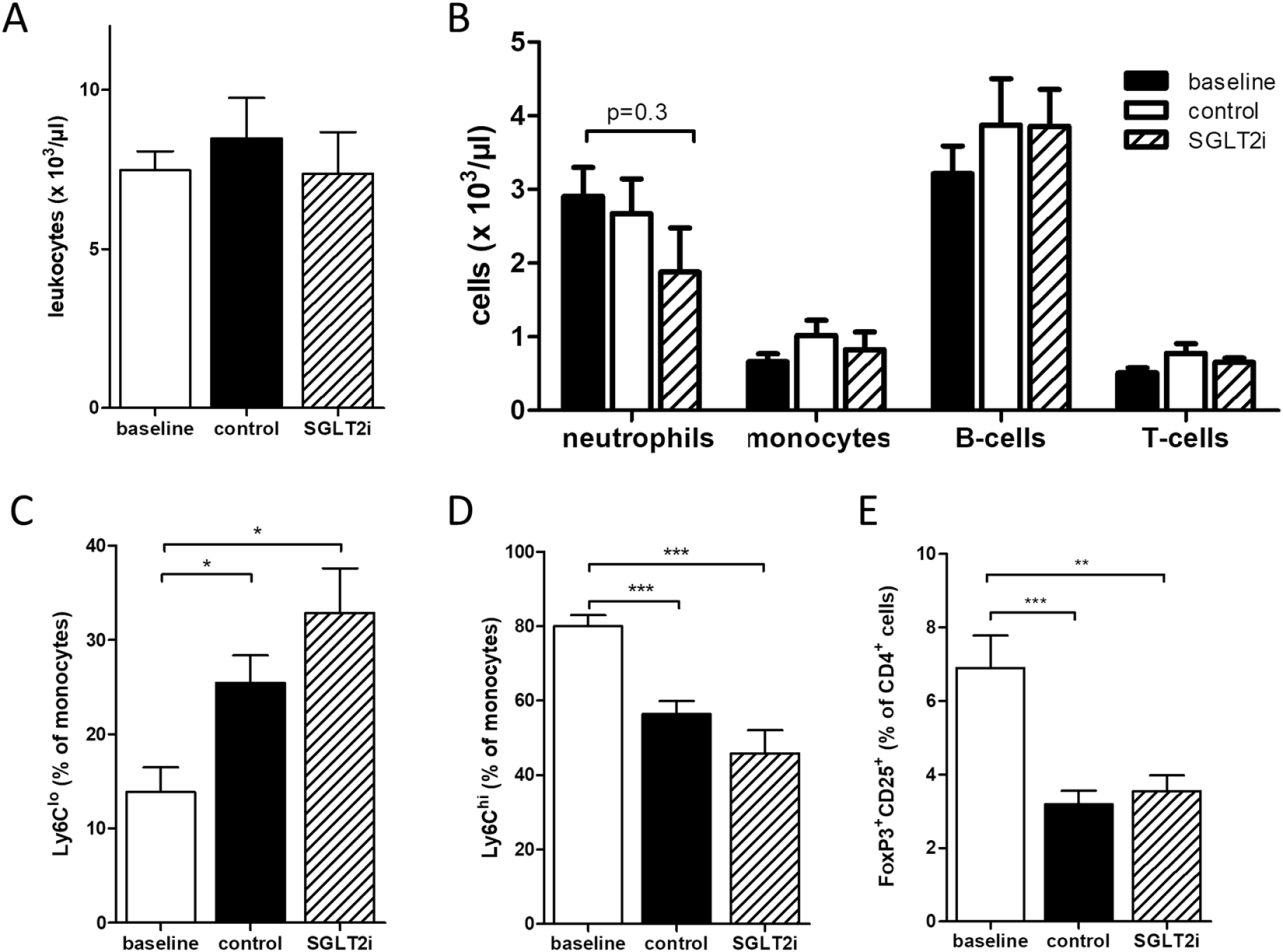
Leukocytes and leukocytes subsets are not affected by the SGLT2-inhibitor empagliflozin during atherosclerosis regression. **(A)** Quantification by flow cytometry of total circulating leukocytes, **(B)** leukocytes subsets, **(C)** patrolling, “non-classical” Ly6C^lo^ and **(D)** pro-inflammatory Ly6C^hi^ monocytes. **(E)** regulatory FoxP3^+^ T lymphocytes. N = 7–9/group. *p < 0.05, **p < 0.01, ***p < 0.001. Error bars represent SEM.

To study possible mechanisms by which glucose lowering accelerates atherosclerotic plaque regression we first analyzed circulating leukocytes. We did not detect a significant difference in total leukocyte numbers, B-/T-lymphocytes, monocytes and neutrophils in any of our study groups (Fig. 4A,B). Inflammatory, Ly6C^high^ monocytes decreased in both regression groups (Fig. 4C,D). However, there was no significant difference in Ly6C^high^ and Ly6C^low^ monocytes between the SGLT2-inhibitor and the control group. These data differ from those reported in a previous study using a different protocol^18^. Regulatory T-cells decreased in both regression groups compared to baseline (Fig. 4E), in line with a recent report showing hypercholesterolemia-dependent regulatory T-cell development^19^. However, there was no difference of regulatory T-cells between the two regression groups. Overall these data suggest that difference in plaque size and composition after glucose lowering are not due to changes in numbers of circulatory cells.

### Glucose lowering by empagliflozin alleviates proliferation of plaque resident macrophages

**Figure 5:**
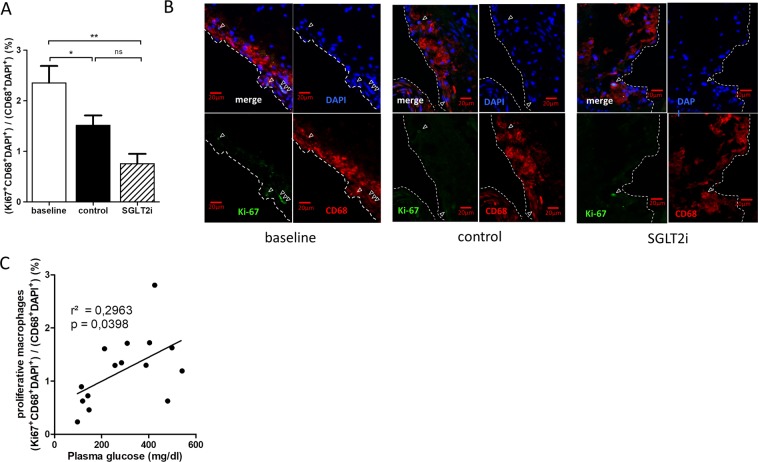
Glucose lowering by the SGLT2-inhibitor empagliflozin alleviates proliferation of plaque resident macrophages. (**A)** Quantification of proliferating Ki67^+^ macrophages in atherosclerotic lesions. N = 7–9/group **(B)** Representative immunofluorescence staining of the aortic root of baseline, control and SGLT2i mice. Proliferating macrophages were stained with anti-CD68 (red), anti-Ki67 (green), and 4’,6-diamidino-2-phenylindole (DAPI; blue) monoclonal antibodies. White dashed line represents border of the atherosclerotic plaque to the vessel lumen. Red scale = 20 µm **(C)** Correlation between plasma glucose and proliferating, plaque-resident macrophages in the two regression groups. *p < 0.05, **p < 0.01. ns = not significant. Error bars represent SEM.

Macrophages contribute substantially to atherosclerosis progression and regression^20,21^. Lesional macrophage content can be reduced in three ways: reduced recruitment into the plaque, emigration out of the plaque and reduced proliferation within the plaque^22^. Recent studies demonstrated that local macrophage proliferation can maintain macrophage accumulation in inflamed^23^ and normal tissues^24,25^. In advanced atherosclerotic lesions, local macrophage proliferation contributes significantly to macrophage accumulation^26^. In our study, proliferating Ki67^+^ macrophages decreased by ~37% with cholesterol lowering compared to baseline. Proliferation was even further diminished in regression mice with normal plasma glucose (~68% compared to baseline, Fig. 5A,B). Furthermore, the number of proliferating macrophages within the atherosclerotic lesions correlated with plasma glucose levels in all regression mice (Fig. 5C). In contrast, there was no correlation of proliferating macrophages with plasma cholesterol levels in the regression groups (data not shown).

### SGLT2 inhibition decreased leukocyte adhesion *in vivo*

**Figure 6:**
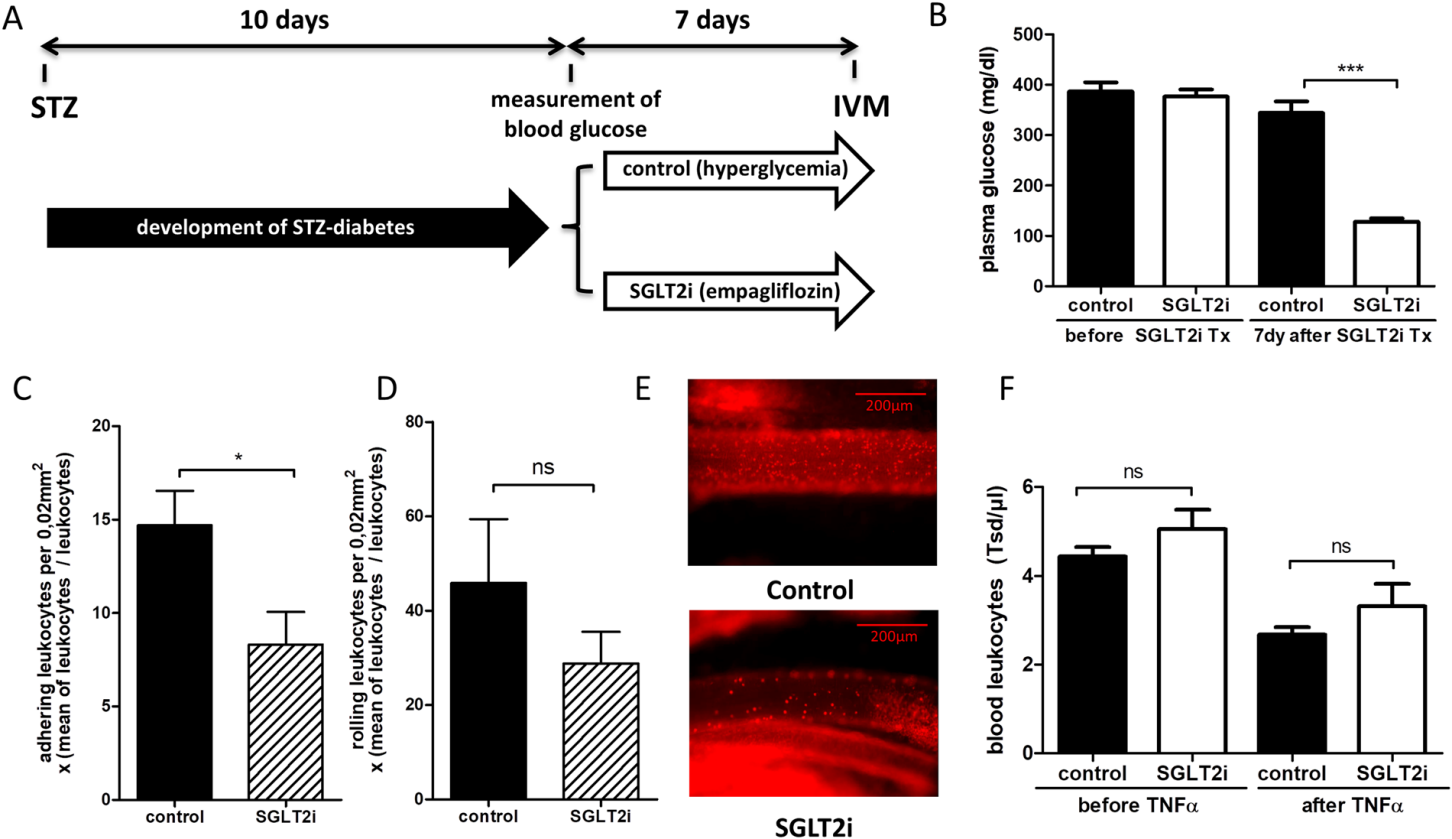
SGLT2 inhibition leads to decreased leukocyte adhesion *in vivo*. (**A)** Timeline of the intravital microscopy (IVM) study. Male BL/6J WT-mice were rendered diabetic by 5 consecutive STZ injections at age 6 weeks. 10 days later 4-hour-fasting-plasma glucose was measured. Mice with glucose levels >250 mg/dl were included into the study. Mice then received either the SGLT2i empagliflozin or control. After one week IVM was performed. **(B)** 4-hour fasting plasma glucose levels ten days after STZ injection (before SGLT2i Tx) and seven days after initiation of SGLTi treatment. **(C)** Quantification of leukocyte adhesion in intestinal venules assessed by intravital microscopy. **(D)** Quantification of rolling leukocytes in intestinal venules by intravital microscopy, (n = 8–10/group). **(E)** Representative still images of videos acquired by intravital microscopy (40x). (**F**) Quantification of blood leukocytes before and after i.p. injection of TNFα for IVM (n = 11–13/group). *p < 0.05, ***p < 0.001. ns = not significant. Error bars represent SEM.

Local macrophage accumulation has long been believed to primarily associate with the recruitment of blood Ly-6C^high^ monocytes to the inflamed vessel wall^27,28^. We assessed recruitment of leukocytes to the vessel wall in STZ-diabetic mice rendered normoglycemic by SGLT2 inhibitor treatment compared to hyperglycemic, vehicle treated mice by intra-vital microscopy (Fig. 6A,B). Leukocyte adhesion to the vessel wall was significantly decreased in normoglycemic mice whereas leukocyte rolling did not reach statistical significance. Again, circulating leukocyte numbers were not different between the groups, thus excluding a confounding factor by increased or decreased leukocyte numbers (Fig. 6C–F).

## Discussion

Regression, or reversal, of atherosclerosis has become an important clinical objective. The use of statins and more recently PCSK9 inhibitors has led to atherosclerosis regression and reductions in vascular events. However, not all patients appear to benefit equally from cholesterol reduction. This is especially apparent in patients with diabetes, who display impaired plaque regression^4^. The aim of this study was to develop a feasible model to study the role of hyperglycemia in diabetic regression. We show that plasma glucose normalization in STZ-diabetic mice accelerates features of plaque regression, likely by impeding leukocyte invasion into the vessel wall and macrophage proliferation.

Over the past two decades different strategies to study atherosclerosis regression in animal models such as aortic transplant and the “Reversa” mouse have been described^29,30^. However, the usefulness of these models is limited by its surgical or genetic complexity. We have recently reported a novel reversible model of atherosclerosis progression and regression using oligonucleotide regulation of the LDLR^8^. This model circumvents many of the challenges associated with other mouse models of regression. To achieve higher plasma cholesterol levels and thus larger atherosclerotic lesions that are comparable to current genetic knock-out mouse models of atherosclerosis (e.g. LDLR- and ApoE-deficient mice) we now used a combination of LDLR and SRB1 ASOs. Others have reported increased diet-accelerated aortic sinus atherosclerosis, myocardial infarction, and early death in SRB1 and either LDLR or ApoE- double knock-out mice^31,32^. In our study, total plasma cholesterol levels increased to ~650 mg/dl after 12 weeks on a HCD and mice displayed substantial atherosclerotic lesions in the aortic root, but we did not observe increased mortality during atherosclerosis progression; myocardial infarction or occlusive coronary atherosclerosis were not assessed. As expected, both plasma cholesterol and triglycerides increased substantially following the induction of insulin-deficient diabetes by STZ injection^15^, replicating diabetic dyslipidemia in humans. After induction of STZ-diabetes we could lower plasma cholesterol and triglyceride levels below thresholds reported to induce atherosclerosis regression in mice^33^.

Previous atherosclerosis studies in diabetic mice show markedly impaired regression compared to controls, despite similar plasma lipid lowering^15,34^. To study the effect of glucose in atherosclerosis regression, we selectively lowered plasma glucose in our diabetic atherosclerosis model by administration of the SGLT2 inhibitor empagliflozin. Empagliflozin, in a randomized, placebo-controlled study, led to a significant reduction in cardiovascular death and hospitalization rate due to heart failure^35^. Unlike many other glucose-lowering therapies, SGLT2 inhibitors act independently of insulin secretion or action^36^. Thus, they are very suitable to selectively study the effect of glucose on atherosclerosis regression in our mouse model.

Several groups have described anti-atherosclerotic effects of SGLT2 inhibitors in atherosclerosis progression in diabetic mouse models^18,37,38^. In contrast, there is only one report to our knowledge that studied the SGLT2 inhibitor dapagliflozin on atherosclerosis regression in diabetic mice^5^. Nagareddy *et al*. showed that treatment of hyperglycemia reduces monocytosis, decreases entry of monocytes into atherosclerotic lesions and promotes atherosclerosis regression. In contrast, we did not detect differences in leukocytes or leukocytes subsets (e.g. monocytes, neutrophils) in our mice. Several factors can explain this difference: First, all our mice (both baseline and regression groups) were diabetic, thus we did not compare to non-diabetic mice. Secondly, leukocytosis is likely affected by the duration of STZ-diabetes, which was 3 weeks shorter in our study. Indeed, we detected significant leukocytosis and monocytosis after prolonged STZ-diabetes and a decline by SGLT2-inhibitor treatment (data not shown). Third, there are substantial differences in the regression models used, for example an antisense/sense oligonucleotide approach with markedly lower plasma cholesterol levels versus a chow diet-switch only or an aortic transplant mouse model. Finally different SGLT2 inhibitors might differ in their effects.

In our study, normoglycemic regression mice showed a significant amelioration of plaque growth compared to hyperglycemic control mice after cholesterol reduction. Furthermore, normoglycemic regression mice displayed even less macrophages and lipid accumulation and more collagen content within atherosclerotic plaques compared to hyperglycemic control mice, suggesting that glucose lowering accelerates features of plaque stability. *In vitro* studies have shown that high glucose levels enhance foam cell formation in cultured macrophages^39,40^. Glycation of lipoproteins has been shown to increase their binding affinity for proteoglycans of the subendothelial extracellular matrix, thereby contributing to lipid accumulation^41^. *In vivo*, atherosclerosis progression studies in non-diabetic and diabetic mice have also shown reduced plaque-resident macrophages after glucose reduction by SGLT2-inhibition^18,37^. However SGLT2-inhibitor treatment in these hyperlipidemic mice also improved plasma lipid profiles, thereby likely obscuring any glucose-specific effect. In contrast, both plasma cholesterol and triglycerides did not differ between our hyperglycemic and normoglycemic mice during regression. The data in humans on the effects of SGLT2 drugs on circulating lipoproteins is also mixed, with some studies showing that these drugs reduce triglycerides and increase HDL as well as LDL, and others showing no effects^42,43^.

The main effect of empagliflozin is reduction of plasma glucose. We cannot fully exclude off-target effects of empagliflozin on atherosclerosis regression but believe it unlikely for the following reasons: First, in a previous study glucose reduction by either SGLT2 inhibition (in this case dapagliflozin) or by SGLT2 antisense oligonucleotide led to similar effects on atherosclerosis and white blood cells in mice^5^. Secondly, expression of the SGLT2 is predominantly confined to the endothelial lining of proximal tubule in the kidneys, with only trace expression in monocytes/macrophages and other tissues^44^ (Supplementary Fig. 2).

Decreased cell recruitment to the atherosclerotic plaque^45^, increased egress of macrophages^46^ and decreased macrophage proliferation^26,47^ are likely to participate in the resolution or regression of atherosclerotic lesions^7^. In line with the latter, we detected less proliferating macrophages in SGLT2 inhibitor treated regression mice and plasma glucose levels correlated positively with intra-plaque proliferating macrophages. In a mouse model of atherosclerosis progression Lamharzi *et al*. have shown that the combination of hyperglycemia and hyperlipidemia stimulates macrophage proliferation, likely by glucose-oxidized LDL^48^. To our knowledge we are first to report decreased intra-plaque macrophage proliferation by glucose reduction in an atherosclerosis regression model. Given the relative low numbers of intra-plaque proliferating macrophages in our and other studies with non-diabetic mice^47,49^, further detailed analyses (e.g. BrdU labeling, gene expression analysis of laser-captures macrophages) will be necessary to evaluate the contribution of glucose-dependent proliferation of intra-plaque macrophages.

In addition to decreased macrophage proliferation, glucose reduction in STZ-diabetic mice also affected leukocyte recruitment to the vessel wall, as shown by intra-vital microscopy. Studies on isolated vascular cells suggest that elevated glucose concentrations cause a plethora of atherogenic responses by generation of advanced glycation end-products (AGEs) or reactive oxygen species that ultimately lead to increased inflammation via activation of nuclear factor κ-B^50^. High glucose exposure induces monocyte activation, monocyte-endothelial cell adhesion and transmigration by increasing adhesion molecules such as VCAM-1 and cytokines such as MCP-1, Interleukin-1β^51–53^. *In vivo*, hyperglycemia increased expression of adhesion molecules on the endothelium, and increased accumulation of leukocytes on the endothelium has been observed in models of STZ- or alloxan-induced diabetes and hyperglycemia^54–56^. Our observation is in agreement with Nagareddy *et al*. who attributed a decrease of plaque resident macrophages to reduced monocyte entry into the vessel wall^5^.

In summary, our study advances the new antisense/sense oligonucleotide-driven approach to investigate atherosclerosis regression in diabetic mice. It obviates the need for the technically demanding use of transplants or the breeding required for regression. Glucose reduction accelerated atherosclerosis regression; possibly by decreased intra-plaque macrophage proliferation and decreased leukocyte recruitment to the vessel wall. Further studies are needed to investigate how glucose mediates these effects *in vivo*.

## Supplementary information

Supplementary information

## Data Availability

All datasets generated during and/or analyzed during the current study are available from the corresponding author on reasonable request.
