# Supplementary material for: Glucose lowering by SGLT2-inhibitor empagliflozin accelerates atherosclerosis regression in hyperglycemic STZ-diabetic mice
Source: Sci Rep. 2019 Nov 29;9:17937. doi: 10.1038/s41598-019-54224-9 (PMC6884628; doi:10.1038/s41598-019-54224-9)

Supplementary Information

Glucose lowering by SGLT2-inhibitor empagliflozin accelerates atherosclerosis regression in hyperglycemic STZ-diabetic mice

Jan Pennig[1], Philipp Scherrer[1], Mark Colin Gissler[1], Nathaly Anto-Michel[1], Natalie Hoppe[1], Lisa Füner[1], Carmen Härdtner[1], Peter Stachon[1], Dennis Wolf[1], Ingo Hilgendorf[1], Adam Mullick[2], Christoph Bode[1], Andreas Zirlik[1,3], Ira J. Goldberg[4], Florian Willecke[1*]

[1]*University Heart Center Freiburg-Bad Krozingen, Cardiology and Angiology I, University of Freiburg, Germany*
[2] *Ionis Pharmaceuticals, Carlsbad, California*
[3] *Division of Cardiology, Medical University of Graz, Graz, Austria*
[4] *Department of Medicine, New York University Langone Health, New York, NY*

Supplementary Figure 1: Hepatic mRNA expression of Srb1 and Ldlr after ASO control or combined LDLR/SRB1 ASO treatment, assessed by quantitative PCR, N = 8 and 11/group, respectively. *** = p<0,001.

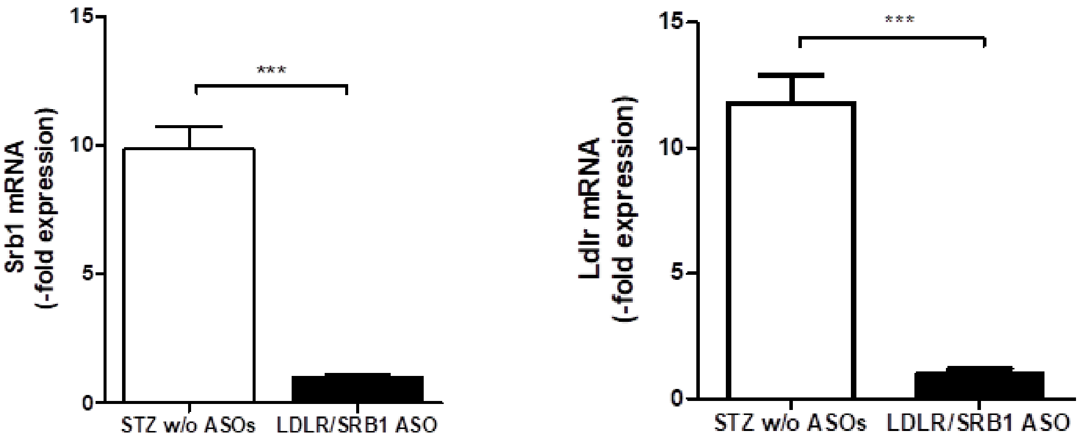

Supplementary Figure 2: Relative expression of Sglt2 (slc5a2) mRNA expression. Data derived from ImmGen RNAseq database

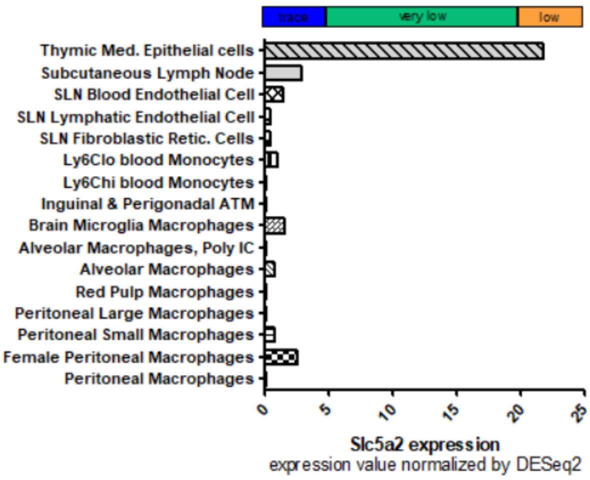

Supplement: Supplementary file 1 — Supplementary information [file 41598_2019_54224_MOESM1_ESM.pdf]
